# MMP-9, TIMP-1 and inflammatory cells in sputum from COPD patients during exacerbation

**DOI:** 10.1186/1465-9921-6-151

**Published:** 2005-12-22

**Authors:** PF Mercer, JK Shute, A Bhowmik, GC Donaldson, JA Wedzicha, JA Warner

**Affiliations:** 1 School of Biological Sciences, University of Southampton, Southampton, UK; 2 Department of Medical Specialties, University of Southampton, Southampton, UK; 3 Academic Unit of Respiratory Medicine, Royal Free and University College Medical School, London, UK

## Abstract

**Background:**

Irreversible airflow obstruction in Chronic Obstructive Pulmonary Disease (COPD) is thought to result from airway remodelling associated with aberrant inflammation. Patients who experience frequent episodes of acute deterioration in symptoms and lung function, termed exacerbations, experience a faster decline in their lung function, and thus over time greater disease severity However the mechanisms by which these episodes may contribute to decreased lung function are poorly understood.

This study has prospectively examined changes in sputum levels of inflammatory cells, MMP-9 and TIMP-1 during exacerbations comparing with paired samples taken prior to exacerbation.

**Methods:**

Nineteen COPD patients ((median, [IQR]) age 69 [63 to 74], forced expiratory volume in one second (FEV1) 1.0 [0.9 to1.2], FEV1% predicted 37.6 [27.3 to 46.2]) provided sputa at exacerbation. Of these, 12 were paired with a samples collected when the patient was stable, a median 4 months [2 to 8 months] beforehand.

**Results:**

MMP-9 levels increased from 10.5 μg/g [1.2 to 21.1] prior to exacerbation to 17.1 μg/g [9.3 to 48.7] during exacerbation (*P *< 0.01). TIMP-1 levels decreased from 3.5 μg/g [0.6 to 7.8] to 1.5 μg/g [0.3 to 4.9] (*P *= 0.16). MMP-9/TIMP-1 Molar ratio significantly increased from 0.6 [0.2 to 1.1] to 3.6 [2.0 to 25.3] (*P *< 0.05). Neutrophil, eosinophil and lymphocyte counts all showed significant increase during exacerbation compared to before (*P *< 0.05). Macrophage numbers remained level. MMP-9 levels during exacerbation showed highly significant correlation with both neutrophil and lymphocyte counts (Rho = 0.7, *P *< 0.01).

**Conclusion:**

During exacerbation, increased inflammatory burden coincides with an imbalance of the proteinase MMP-9 and its cognate inhibitor TIMP-1. This may suggest a pathway connecting frequent exacerbations with lung function decline.

## Background

Chronic obstructive pulmonary disease (COPD) is a classic disease of airway damage and remodelling, characterised by slowly progressive airflow obstruction, resulting in increasing dyspnoea and exercise limitation. It is a widely accepted theory that an important causative factor is extracellular matrix (ECM) remodelling, resulting from aberrant inflammation and disruption of the proteinase – antiproteinase balance; reviewed [1]. The identity of candidate proteinases has been a subject of much debate, however the balance between matrix metalloproteinases (MMPs) and their inhibitors; the tissue inhibitors of metalloproteinase (TIMPs), is thought to be important in pathogenesis [1-3].

MMPs are a family of up to 26 homologous endopeptidases, which can collectively degrade all of the protein components of the extracellular matrix [4]. They are produced by a range of stromal cells and by two of the major inflammatory cells implicated in COPD – neutrophils and alveolar macrophages [5,6]. Recent studies have shown that levels of MMPs, especially MMP-9, are elevated in the bronchial alveolar lavage (BAL) fluid from patients with COPD, compared to normal controls [5,7], and high levels of both MMP-9 and its cognate inhibitor TIMP-1 have been found in sputum from chronic bronchitics [8] and correlated with decrease in lung function [9,10]. While these studies focused on stable COPD, there has been no analysis to date of MMP-9 and TIMP-1 variation during exacerbations.

Patients with moderate to very severe COPD are prone to frequent exacerbations, which are associated with a poorer health-related quality of life [11]. Exacerbations can be triggered by viral or bacterial infection of the lower respiratory tract [12,13] both of which increase airway and systemic inflammatory response [14-16]. Exacerbations also contribute to the accelerated decline in FEV1 that is characteristic of COPD [17,18].

Using a well monitored group of COPD patients, this study has prospectively investigated changes in sputum levels of MMP-9 and TIMP-1 during exacerbations in COPD and compared these to samples taken from the same individuals prior to exacerbation. The aim of the study was to see whether the MMP/TIMP balance was upset during exacerbation as this might suggest a pathway between exacerbations, ECM remodelling and lung function decline.

## Methods

### Subjects

Nineteen patients were recruited from a cohort of patients followed since October 1995 by the East London COPD study [14,19]. Inclusion criteria and enrolment for the study have been previously published [14,19].

The patients were recording on diary cards each morning any increase over their normal, stable condition of (i) dyspnoea, sputum purulence or sputum volume (major symptoms) and (ii) colds (nasal discharge/congestion), wheeze, sore throat, cough and fever (minor symptoms). This was a yes/no answer. Exacerbation onset was identified as the first of two or more consecutive days with increase in either two or more major symptoms, or any one major symptom plus any minor symptom. The prospective data collected on the diary cards before and at onset of the exacerbation showed that peak expiratory flow fell from 246 l/min (223 to 313) averaged over days 14-8 preceding exacerbation by 7.1 l/min (-22 to 3) on the day of onset. Similarly, the number of symptoms rose from zero to 2 (2 to 3) on the day of onset. Patients with any deterioration of symptoms were encouraged to contact the clinical team by telephone, and were seen in clinic for sputum sampling, generally within 48 hours. Patients also attended 3 monthly clinics at which samples were obtained when the patient was exacerbation free (clinically stable).

19 individuals (Table 1) were recruited and gave informed, written consent. 12 of the 19 samples taken at exacerbation were paired with a prospectively collected stable sample obtained within a median of 4 months (IQR 2–8 months) prior to the sampled exacerbation. The study had ethics approval from the Ethics Committee of the East London and City Health Authority.

**Table 1:** Characteristics of the 19 patients in the study

	Median	IQR
Age, yr	69	63 to 74
FEV_1_, L (stable)	1.0	0.9 to 1.2
FEV_1_, %	37.6	27.3 to 46.2
FEV_1_/FVC	45.2	25.9 to 54.8
pO_2 _(kPa)	9.27	8.45 to 9.51
pCO_2 _(kPa)	5.66	5.49 to 6.36
O_2 _saturation breathing room air (%)	94	92 to 95
Reversibility to salbutamol (%)	9.7	5.0 to 13.5

	%	

Males	85	
Ex smokers	60	
Current smokers	40	
Daily taking oral steroids	5	
Daily taking inhaled steroids	90	

### Sputum preparation

Sputum samples were processed as per Bhowmik *et al *2000 [14]. Briefly, the sputum was incubated with four times its weight of 0.01 M DTT in HBSS, at 4°C for 15 minutes. Sputum dilution was in proportion to the weight collected thus standardizing between time points and patients the supernatants used for assay of proteinases and other markers. The volume of HBSS was then doubled (ten fold dilution of original sputum sample) and incubated for a further 5 minutes. The suspension was then filtered through 50 μm nylon gauze to remove mucus and debris without removing any of the cells and centrifuged at 790 g for 10 minutes. The cell free supernatant was removed and stored at -70°C. The cell pellet was resuspended. The total cell count was determined with a Neubauer haemocytometer using the trypan blue exclusion method to determine cell viability, blue cells were counted as non viable. Cytospins were made from the cell suspension and stained with Diff-Quik to obtain a differential cell count.

### Zymography

**Figure 1:**
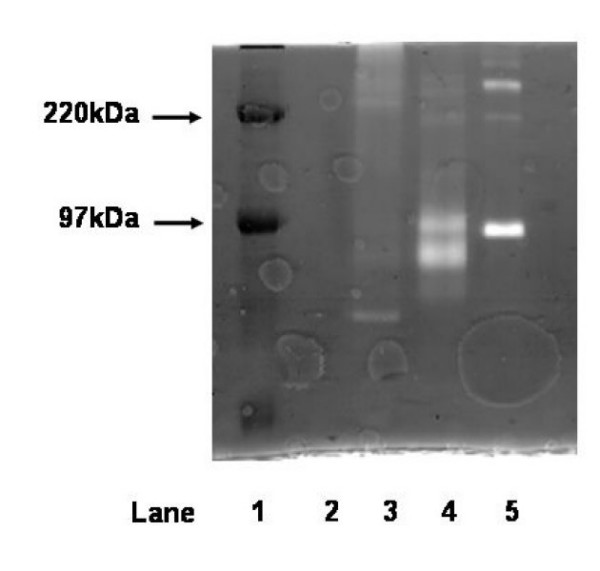
**Sample zymogram showing immunoprecipitation of MMP-9 from sputum**. 10 μl volumes loaded per lane. Lane 1: High range molecular weight markers (Amersham Biosciences); lane 2: polyclonal antiMMP-9 (4 μg/ml); lane 3: exacerbation sputum sample (1:100 dilution) + antiMMP-9 (4 μg/ml); lane 4: exacerbation sputum sample (1:100 dilution); lane 5: pro-MMP-9 standard (92 kDa) (100 ng/ml).

Quantitative gelatin zymography was carried out as described by Kleiner et al [20] to analyse MMP-9 levels. Briefly, samples were prepared by dilution into a 5X zymography sample buffer consisting of 0.4 M Tris, pH 6.8, 5% SDS, 12.5% sucrose, and 0.03% bromophenol blue. 10 μl volumes of sample were loaded onto a 7.5% polyacrylamide gel containing 0.1% gelatin and the proteins separated by electrophoresis. The gel was washed twice in buffer containing 20 mM Tris, and 2.5% Triton, pH 7.8 and incubated overnight at 37°C in Tris/Triton buffer supplemented with 10 mM CaCl2 and 5 μM ZnCl. The following day the gels were stained with 0.5% Coomassie and the gelatinases visualised as clear areas on a dark blue background (see Figure 1). The major band of MMP-9 activity in all samples was proMMP-9 observed at 92 kDa, this accounted for a median 92% of total MMP-9 activity (total n = 31 samples, IQR 78–100%). Additional bands were also observed at 125 kDa and 220 kDa comprising MMP-9 complexed with inhibitors and MMP-9 dimers. In a small minority of samples active MMP-9 was observed below the major MMP-9 band at 82 kDa. MMP-2 activity was found to migrate to 72 kDa. The identity of MMP-9 was further verified by immunoprecipitation with a polyclonal antibody to MMP-9 (4 mg/ml, Biogenesis. Dorset, UK) Figure 1.

Band intensity was captured and digitised by scanning densitometry (using scan analysis). The optical optical density for each band of interest was calculated as area under curve. This value was converted to total MMP-9 for each patient by combining the values for each band (220, 125 and 92 kDa) and normalizing to MMP-9 standard (1 ng/lane, Biogenesis. Dorset, UK) (see Figure 1). Samples outside of the accurate limit of detection were diluted up to a hundred fold and rerun.

### Analysis of TIMP-1

Samples were analysed using a commercial Biotrak^® ^TIMP-1 ELISA (Amersham Biosciences. Bucks. UK.) specific for both total free TIMP-1 and TIMP-1 complexed to MMPs. ELISAs were run according to the manufacturer's instructions.

### Statistical analyses

All differences within sets of paired data were analysed using the non-parametric Wilcoxon signed rank test. Correlations were calculated using the non-parametric Spearman correlation. *P *value < 0.05 was considered significant.

## Results

### Levels of MMP-9 and TIMP-1 in paired samples

**Figure 2:**
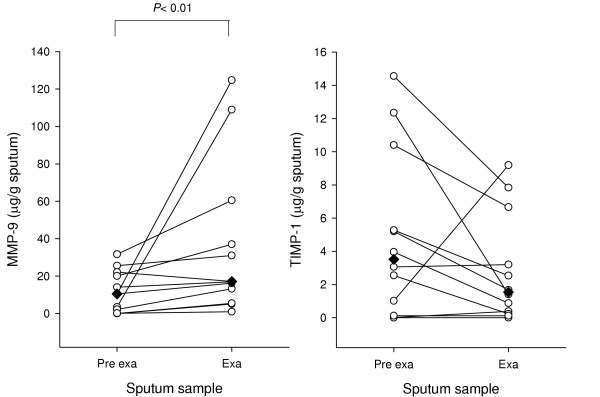
**MMP-9 and TIMP-1 levels in sputum from COPD patients**. Samples were taken from patients prior to and during exacerbation (n = 12). MMP-9 was measured by gelatin zymography and TIMP-1 was measured by ELISA and corrected for weight of sputum. Individuals are shown as open circles and median values as diamonds. Data were analysed by non parametric Wilcoxon signed rank test.

Figure 2 shows that total MMP-9 levels increased from a median of 10.5 μg/g (IQR 1.2 to 21.1) prior exacerbation to 17.1 μg/g (IQR 9.3 to 48.7) during exacerbation (*P *< 0.01) (n = 12). Additionally, the percentage of samples showing an active MMP-9 band (running at 82 kDa) was observed in 1 paired sample prior to exacerbation compared to 4 during exacerbation (data not shown). Of the total of 19 samples taken during exacerbation, 7 showed an active MMP-9 (82 kDa) band.

**Figure 3:**
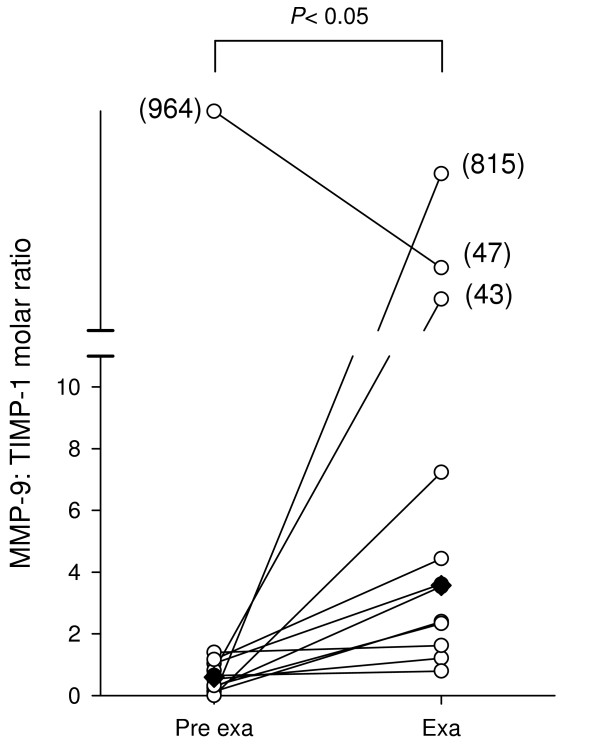
**Molar ratio between MMP-9 and TIMP-1 in COPD sputum**. The data from figure 2 was used to calculate a molar ratio between MMP-9 and TIMP-1. Median values are marked as diamonds. Data were statistically analysed by a non parametric Wilcoxon signed rank test.

In contrast, median levels of TIMP-1 decreased from 3.5 μg/g (IQR 0.6 to 7.8) to 1.5 μg/g (IQR 0.3 to 4.9), with this change approaching statistical significance (*P *= 0.16). Proteinase/antiproteinase imbalance at exacerbation (Figure 3) is reflected by a change in the MMP-9/TIMP-1 molar ratio from a median of 0.6 (IQR 0.2 to 1.1) before to 3.6 (IQR 2.0 to 25.3) during exacerbation (*P *< 0.05). Levels of MMP-2 were also analysed by gelatin zymography, however out of all sputum samples analysed only one showed a small amount of MMP-2 activity (data not shown).

### MMP-9 and cellular inflammation

**Figure 4:**
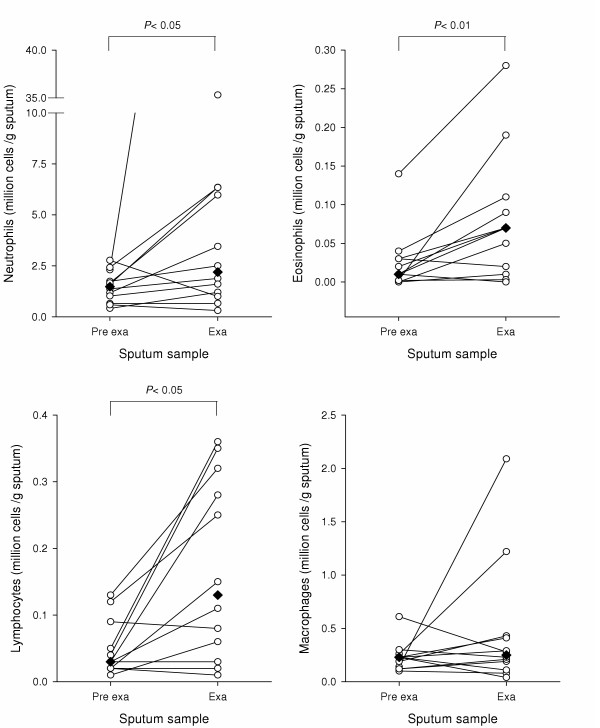
**Inflammatory cell counts prior to and during exacerbation**. Differential neutrophil, eosinophil, lymphocyte and macrophage counts were carried out on samples from COPD patients and corrected for weight of sputum. Samples were taken from patients prior to and during exacerbation (n = 12). Median cell counts are marked as diamonds. Data were analysed by a non parametric Wilcoxon signed rank test.

Figure 4 indicates that total cell influx into sputum increases significantly during exacerbation (*P *< 0.05). Figure 4 and table 2 show that neutrophil, eosinophil and lymphocyte numbers all increase significantly during exacerbation, compared with pre exacerbation levels. In contrast macrophage numbers remained unchanged and show a significant decrease as a proportion of total cells.

**Table 2:** Total and differential leukocyte cell counts in COPD sputum sampled prior to and during exacerbation† (n = 12)

Cell type	Pre-exacerbation		Exacerbation		*P*-value
	Median	IQR	Median	IQR	

Total cell (10^6 ^cells/g)	1.61	0.88–2.25	2.86	1.64–7.2	<0.01
% viability	65.14	56.05–74.92	83.33	65.57–88.60	<0.01
Neutrophil (10^6 ^cells/g)	1.48	0.84–2.02	2.19	1.10–6.16	<0.05
% Neutrophils	80.0	72.63–89.30	87.5	78.38–89.25	NS
Macrophage (10^6 ^cells/g)	0.23	0.13–0.25	0.26	0.15–0.42	NS
% Macrophages	17.25	6.63–22.25	6.0	5.38–17.5	<0.05
Eosinophil (10^6 ^cells/g)	0.01	0.002–0.03	0.07	0.02–0.10	<0.01
% Eosinophils	0.75	0.25–1.38	1.0	0.63–3.00	NS
Lymphocyte (10^6 ^cells/g)	0.03	0.02–0.07	0.13	0.05–0.3	<0.05
% Lymphocytes	2.50	1.38–4.38	4.5	1.25–5.63	NS

† Paired sputa from 12 individuals.

Percentage squamous cell contamination of sputum varied from 5.14% (IQR 1.81–6.20) during stable COPD, to 1.6% (0.43–4.58) during exacerbation (data not shown).

**Figure 5:**
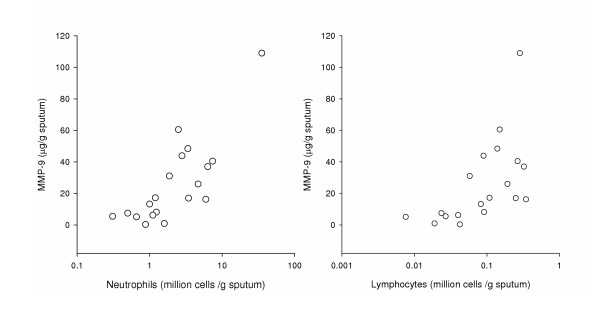
**Relationship between cell infiltration and MMP-9 in COPD sputum during an exacerbation**. Neutrophil (Left hand panel) and lymphocyte (right hand panel) counts were carried out on sputum samples from COPD patients during exacerbation (n = 19), and corrected for weight of sputum. Measurement of MMP-9 levels was carried out on cell free sputum supernatants. Cell count data were expressed as million cells/g (log scale) and MMP-9 data as μg/g.

Figure 5 and table 3 show that during exacerbation (n = 25) MMP-9 correlates significantly with levels of both neutrophils and lymphocytes (Rho = 0.7,*P *< 0.005 for both cell types). Correlation between MMP-9 and macrophages or eosinophils did not reach the same level of significance (Rho = 0.5 *P *< 0.1 for macrophages and Rho = 0.5 *P *< 0.05 for eosinophils). No relationship was found between MMP-9 and any cell type in any of the paired sputa obtained prior to exacerbation when the patient was clinically stable (Data not shown).

**Table 3:** Spearman's correlation between MMP-9 level and inflammatory cell counts during exacerbation† (n = 19)

Correlation	Rho	P value
Neutrophils	0.74	<0.005
Macrophages	0.46	<0.1
Eosinophils	0.45	<0.05
Lymphocytes	0.72	<0.005

† Sputa obtained from 19 exacerbations

## Discussion

This is the first pilot study to look at how levels of the inflammatory proteinase, MMP-9 and its cognate inhibitor, TIMP-1 change in the airway during an exacerbation. The results indicate that exacerbations are associated with an increase in MMP-9 burden in the airways. This increase could result in ECM destruction in the airways and contributing to airway remodelling and the decline in lung function seen in COPD patients. Previous studies have related MMP-9 to parenchymal destruction and lung function decline, in sub clinical [5] and established emphysema [7]. Studies with induced sputum from stable COPD patients have found that levels of MMP-9 and TIMP-1 are elevated in COPD patients compared to controls [9,10]. Levels were found to correlate with both increased neutrophilia and decrease in lung function. These studies suggest that an underlying proteolytic environment exists in the airways in COPD, however studies where molar ratio of MMP-9 to TIMP-1 was analysed found that TIMP-1 levels outweighed MMP-9 [8,21]. Our current study indicates that prior to exacerbation MMP-9/TIMP-1 balance favours the inhibitor. During exacerbation however MMP-9 levels significantly increase, tipping the balance in favour of MMP-9. A similar finding has been reported in asthmatic exacerbations which found that circulating levels of MMP-9 were increased in patients during exacerbation compared with convalescence [22]. The frequency of shift between a "proteolytic" phenotype during exacerbation and "fibrotic" phenotype outside exacerbation, may be an important driving factor in airway remodelling, and deserves further research.

Our data show that number of neutrophils, eosinophils, and lymphocytes all increase in the airways during an exacerbation, whilst levels of macrophages remained constant. Recent studies have shown that alveolar macrophages from smokers increased production of MMP-9 in response to cigarette smoke conditioned medium [23]. Pro-inflammatory stimuli such as IL-1β and LPS also increased MMP-9 production, leading the authors to suggest that Alveolar Macrophage release of MMP-9 could have relevance in COPD exacerbations. In this present study, a strong correlation was observed during exacerbation between increased neutrophil number and MMP-9 while a weaker association existed between macrophages and MMP-9. While the contribution of macrophage derived MMP-9 cannot be ruled out, it is tempting to speculate from these data that neutrophil influx is the primary source of increased MMP-9 during exacerbation. The stimulus for increased neutrophil influx was not assessed in this study, however it seems likely that neutrophil specific chemokines such as IL8 are involved this response. Recently, Bhowmik *et al*, using sputa from the East London COPD study, reported that levels of IL8 during exacerbation were related to neutrophil number [14].

Additionally, it is of note that the increase in MMP-9 during exacerbation also shows a very close association with increase in lymphocyte number. It is unclear whether these events are related; however it is pertinent to suggest that the increase in neutrophil and lymphocyte numbers is linked. In support of this concept, a recent study in mice has shown that intranasal stimulation by the cytokine IL17, produced by CD4+ T-lymphocytes induced a pronounouned accumulation in both pro and active MMP-9 associated with local accumulation of neutrophils [24]. A recent study by Zhu *et al *reported an increase in the number of CD4+ lymphocytes in the airways during exacerbation in COPD [25]. The authors suggested that virally induced upregulation of the chemokine RANTES during exacerbation could contribute to this. In addition an increased airway eosinophilia, also attributed to RANTES upregulation, was observed.

While eosinophils do not have a clearly defined role in COPD, an increased airway eosinophilia during exacerbation has now been observed in a range of studies including our own [16,25]. The role of eosinophils in disease progression may therefore deserve further investigation. Previous studies using asthmatic BAL identified eosinophils as important sources of MMP-9 [26]. However a comparatively weak, albeit significant association between MMP-9 and eosinophils in our study, suggests eosinophils are not the primary source of MMP-9 during COPD exacerbation.

## Conclusion

We report here that during an exacerbation in COPD there is an increased MMP-9 burden in the airways, which combined with unchanging TIMP-1 leads to MMP-9/TIMP-1 imbalance in favour of MMP-9. Increase in MMP-9 correlates with influx in both neutrophils and lymphocytes and to a lesser extent eosinophil and macrophage numbers. The cross talk between these cells during exacerbation and their contribution to airway remodelling requires further detailed investigation.

## Competing interests

The author(s) declare that they have no competing interest.

## Authors' contributions

PFM assisted in the study design, carried out the zymography and TIMP-1 immunoassays and drafted the manuscript. JKS assisted in study design and interpretation of data. AB collected the sputum samples and carried out the cell counts. GCD participated in the foundation of the patient cohort and advised on the statistical analysis and interpretation of data. JAW^3 ^founded the patient cohort, participated in the study design and coordination and assisted in drafting the manuscript. JAW^1 ^conceived of the study, supervised study design and assisted manuscript drafting. All authors read and approved the final manuscript.
